# Emergency contraception: Awareness, attitudes and barriers of Saudi Arabian Women

**DOI:** 10.12669/pjms.316.8127

**Published:** 2015

**Authors:** Syed Irfan Karim, Farhana Irfan, Norah Al Rowais, Basma Al Zahrani, Riaz Qureshi, Bedoor H Al Qadrah

**Affiliations:** 1 Dr. Syed Irfan Karim, FRCGP. Assistant Professor & Consultant Family Medicine, Deputy Director Family Medicine Residency Training, Department of Family and Community Medicine, College of Medicine, King Saud University, Riyadh, Saudi Arabia; 2 Dr. Farhana Irfan, MRCGP. Assistant Professor, Dept. of Family and Community Medicine, King Saud University Chair for Medical Education Research and Development, College of Medicine, King Saud University, Riyadh, Saudi Arabia; 3 Dr. Norah Al Rowais, SBFM. Associate Professor & Consultant Family Medicine, Department of Family and Community Medicine, College of Medicine, King Saud University, Riyadh, Saudi Arabia; 4 Dr. Basma Al Zahrani, SBFM. Senior Registrar, Department of Family and Community Medicine, College of Medicine, King Saud University, Riyadh, Saudi Arabia; 5 Prof. Riaz Qureshi, FRCGP. Distinguished Professor Family Medicine, Department of Family and Community Medicine, College of Medicine, King Saud University, Riyadh, Saudi Arabia; 6 Dr. Bedoor Hassan Al Qadrah, MBBS. Resident, Department of Family and Community Medicine, College of Medicine, King Saud University, Riyadh, Saudi Arabia

**Keywords:** Emergency Contraception, Awareness, Family Physician, Saudi Arabia

## Abstract

**Objectives:**

To assess knowledge, attitude, and barriers about emergency contraception (EC) among married women of child bearing age.

**Methods:**

A quantitative cross-sectional study was conducted over a 6-month period, commencing in March 2013 at Family Practice Clinics of King Khalid University Hospital (KKUH), Riyadh, Saudi Arabia. Data was collected using a structured pretested questionnaire and analyzed using SPSS version 21.0 statistical software.

**Result:**

A total of 242 women were enrolled in the study. Only 6.2% (15/242) had some knowledge of EC and of these only two had ever used it. Health care professionals were the least reported source of EC information (6.6%, n=1). Majority (73.3%) had negative attitude toward EC being available over-the-counter without a prescription. The most common barriers to using EC were concerns about possible health effects. Only two women (13.3%) considered religious belief as a major hindrance to its use.

**Conclusion:**

Awareness of emergency contraception is very low among women of Saudi Arabia. Health care professionals were the least reported source of information, which is a cause for concern. Our findings reveal an urgent need to educate women about EC, keeping in view the social norms and the Islamic values.

## INTRODUCTION

Unintended pregnancy (both unplanned and unwanted) is a common public health problem worldwide.^1^ It is estimated that in the Middle East and North Africa (MENA) region, one in four pregnancies are unintended, leading to unsafe abortions and jeopardizing the health and well-being of women and their families.^2^,^3^ Emergency contraception (EC) can play a vital role in preventing unintended pregnancies. Over the past many years contraceptives are available in the Arab region,^4^ however, emergency contraception availability and advice is sparse and not very commonly used.

The plethora of research on women’s attitudes and barriers regarding EC use shows that the majority of studies were carried out in the West,^5^-^7^ only few studies have been conducted in the developing countries, especially the Muslim world.^8^-^10^ Relatively, there is sparse information on the reproductive behavior of women in the Arab world, especially Saudi Arabia. It is a pressing problem as unintended pregnancies are not restricted by geographical boundaries. Several factors can influence a person’s attitude, beliefs and conviction regarding a chosen behavior.^11^ Hence, a better understanding of the culturally specific major factors affecting the use of EC needs to be addressed. The current study was designed to assess the knowledge, attitude, and barriers about EC among married women of child bearing age who attended a Family Medicine clinic in Riyadh, a major and capital city of Saudi Arabia.

## METHODS

### Study population and methods

Women visiting the outpatient clinics at the Primary Care Clinics of King Khalid University Hospital (KKUH), Riyadh, Saudi Arabia were invited to participate in this study during the period from March 2013 till Sept 2013.

The sample size was calculated with an estimated prevalence of knowledge about EC assumed to be 25% on the basis of the findings of a previous study in Egypt.^10^ The calculated sample size to achieve a precision of ± 5% was 289. A convenient sampling method was used in this study as no previous studies are available in Saudi Arabia.

The original inventory in English was translated into Arabic. A preliminary pilot study was conducted on 10 women. Women in the reproductive age (18-55 years) were eligible to participate in the study. The age of the participants was recorded and identified as the younger age group (18–34 years) and the older age group (35–55 years). However, unmarried women were not included due to cultural reasons and their shyness to answer questions related to sex.

Data was collected using a two part questionnaire. The first part consisted of socio-demographic data such as age, education, family income, marital status, and number of children. The second part included questions regarding knowledge, the attitude and barriers to EC use. Knowledge was defined according to the participants’ response to the question: “if a woman has unprotected sex, is there anything she can do in the first three days after intercourse that will prevent pregnancy”? Those who said “yes” were considered to have knowledge, while those who said “no” were considered as not having any knowledge of EC. For those who said that they “didn’t know” or gave an ambiguous answer were also considered not to have a knowledge of EC. Those who had knowledge were asked what can she do to prevent pregnancy and what is the correct timing for its use? Had they ever used it in the past? What is the risk of pregnancy at present (using or not using contraception)? And what is the source of their knowledge about EC?

Attitude is an underlying inclination to respond to something favorably or unfavorably. An ABC model of attitudes was used to cover the three domains [affective (feelings), behavioral (behavior) and cognitive (beliefs)]. Likert-scale questions were used to elicit whether women agreed, disagreed or were unsure with five statements regarding their beliefs and attitudes. The following questions covered the three domains,

1. Affective: “Should EC be more widely advertised?”, “Should EC be available without prescription?”, “Would you feel shy to ask for EC?”
2. Behavioral: “EC reduces the chance of pregnancy by up to 75%, would you use it to prevent pregnancy?”
3. Cognitive: “what are the reasons for not using EC?”
  - Religion.
  - Medical
  - Others.

Statistical analysis were performed using Statistical Package for Social Sciences version 21. Descriptive statistics was used to calculate percentages and frequencies. Participants were assured confidentiality and anonymity.

### Ethical statement

The study was approved by the Institutional Review Board (IRB) of KKUH, King Saud University.

## RESULTS

The mean age of the participants was 37.85±10.62 SD years. The demographic characteristics of the respondents are presented in Table-I. The response rate was 84%. Of all the surveys collected, 242 questionnaires were finally analyzed as the remaining were excluded due to incomplete information given by the participating women. Overall, the majority (93.8%) had no knowledge of EC, only 15(6.2%) stated that something can be done after unprotected sex to prevent pregnancy. (Table-II)

**Table-I:** Socio demographic characteristics of the study population.

	No. (n=242)	Percentage
Mean age in years	37.85±10.62	
Education		
Uneducated	21	8.6%
Primary education	55	23.0%
High School	73	30.1%
Graduate	88	36.3%
More	5	2.06
Occupation		
House Wife	177	73.1%
Working women	65	27.0%
Number of Children		
None	26	10.7%
One	41	16.9%
Two	28	11.6%
More than Two	147	60.74%

**Table-II:** Emergency Contraception Question 1: If a woman has unprotected sex is there anything she can do to prevent pregnancy? (n=242).

Response	Respondents	Percentage
Yes	15	6.2%
No	175	72.3%
Don’t know	52	21.5%

Of the 15 women who answered that there was something a woman could do to prevent pregnancy after unprotected sex, seventy three percent were in the older age group, while 27% were less than thirty five years of age. Examination of the responses by age yielded some differences. When they were asked what could be done? Women in the older age group relied more on herbal remedies and other methods, while the knowledge of EC was prevalent in the younger women aged less than 35 years. Natural Herbal remedies was the most frequent method mentioned by 33% of women, followed by extra or high dose birth control pill (13.3%), abortion (13.3), routine birth control pill (6.6%) or other methods like home remedies (20.0%). Only 13.3% of the 15 women mentioned emergency contraception pill (ECP) as a preferred method. None of the participants were aware of the post coital insertion of an IUCD as a method for emergency contraception.

Out of the fifteen women who knew about the EC method, only two (13.3%) had used emergency contraceptive pills previously. The majority (53%, n=8) were not willing to use it at all even if it reduces chance of pregnancy. Only 27% would consider using EC pill if the need arose. The majority (73%, n=11) of these 15 women had used reversible methods of contraception. Out of all the women who were at risk of pregnancy by not using contraception at all or using reversible methods of contraception, only three (20%) had a family planning visit in the past year.

Most participants had received information about EC from their family members (60%, n = 9); social media (20.0%, n=3) and friends (13.3%, n=2) and family planning provider/doctors (6.6%, n=1). (Table-III).

**Table-III:** Source of knowledge about ECP.

	N=15	%
Family members	9	60
Social media (TV/magazine/internet)	3	20
Friends	2	13.3
Doctors/family planning provider	1	6.6

About 73.3% of the total participants had favorable attitudes towards public awareness sessions related to the EC. Increased availability and making ECP an over-the-counter product was rejected by most participants (73%). Only 4 participants welcomed the prescription free status of the ECP. Concerning the decision to use EC by the 15 women with the knowledge of EC, eighty percent (n=12) believed in a mutual decision. A small number of these women (20%, n=3) expressed feelings of shyness as a barrier to purchasing an EC. Worries about medical side effects and health aspects were expressed as a major hindrance to its use (73.3%). Religious belief was not perceived as an important factor as a barrier, as only13.3% (n=2) women stated that they had objection due to religious values. (Table IV)

**Table-IV:** Participants attitudes and beliefs regarding EC (n=15).

Domain	Statement	Agree	Disagree	Unsure
		N (%)	N (%)	N (%)
Affective	Should EC be more widely advertised?	11(73.3)	3(20.0)	1 (6.6)
	Should EC be available without prescription?	4(26.6)	11(73.3)	0 (0)
	Would you feel shy to ask for EC?	3(20.0)	11(73.3)	1(6.6)
Behavioral	EC reduces the chance of pregnancy by up to 75%, would you use it to prevent pregnancy?	4(26.6)	8(53.3)	3(20.0)
Cognitive	What are the reasons for not using EC?			
	Religion.	2(13.3)	13(86.6)	0 (0)
	Medical.	11(73.3)	2(13.3)	2(13.3)
	Others (financial or difficulty to access)	2(13.3)	0(0)	13(86.6)

## DISCUSSION

The awareness of EC among women (6.2%) in this study is very low. This conjures a snapshot of the current knowledge level of Saudi women, which is lower than other Muslim countries.^10^ While it is widely believed that the knowledge of contraception has increased in the western world,^12^ the results of this study show that both the awareness and the use in the current study are low, which is consistent with a previous study.^13^

Inadequate knowledge about contraceptive methods has been cited as one of the major reasons for the lack of its use amongst women.^14^ This can be a product of culture, insufficient counseling, and may even be a reflection of social norms. The level of awareness amongst Saudi women is quite similar to what was found by Marafie.^15^ A possible reason for this could be similar background, status, living conditions and the region. Literature reveals that the significant determinants of the use of contraceptives are younger age, women’s working status and education.^9^,^16^ The findings of this study indicate that older women were less knowledgeable about EC than younger women. This may be because the younger generation is more educated and better exposed to social media. However, an overall pattern of partial knowledge was observed throughout the responses regarding the time frames, types and effectiveness of EC. These findings are consistent with some other studies.^16^,^17^ The lack of proper knowledge about EC could be attributed to a number of factors, such as the family members as being the main source of information, followed by social media and friends. This finding is consistent with previous literature related to this topic.^16^ Another possible reason for inadequate use of the EC could be unawareness due to minimal educational resources available in Arabic language,^18^ as it was found that after an explanation of the method and its effectiveness, many women showed interest in using it in the future if need arises. This indicates that intention and knowledge are good predictor of individual behavior.^11^

As with previous research,^16^,^17^,^19^ in the current study, health care providers and family planning services were rarely a source of information regarding EC. This is of concern and unfortunate, as these health care workers can be source of reliable information related to EC. A possible reason could be inhibition towards sexuality related consultations, which are particularly difficult in some developing countries. As mentioned by Ahlam, physicians in Saudi Arabia tend to avoid initiating discussions about sexual matters in their clinical practices, to respect the cultural norms.^17^ Dissemination of the information about options for contraception should become a part of routine counseling, as knowledge-based barrier is present amongst women for the use of EC.^17^

The results of this study give a mixed picture of attitudes towards EC. The majority of participating women were not prepared to use the EC method. According to the theory of Reasoned Action^11^ the behavior and negative attitude could be two important predictors of not using EC in the future. Most women had negative attitudes towards increased availability and prescription free status of the drug. The major barriers to the EC expressed by women were worries about side effects and health consequences, a result consistent with reports from other countries.^14^,^20^ Additional barriers identified were feeling of shyness and embarrassment about discussions related to contraception. While the reasons for this response could be many, one explanation could be the way women are raised in a Saudi Community.^17^ A helpful strategy to overcome this barrier can be to address the main socio cultural concerns and offer services of female staff for providing education related to the sexual health problems. This is an important consideration for health authorities, as it reflects a need for support and counseling.^21^

It has been well documented in the literature that cultures and religions tend to shape ideas regarding contraception.^22^,^23^ It is surprising and noteworthy, that in the current study, socio- cultural variable such as religion was not perceived as an important factor by the majority of the participants for influencing their use of EC. A rapid change in the Saudi society over the last decade, especially women’s education, changing fertility beliefs and behaviors could explain the evolution of these variables.^16^

In order to explore a woman’s level of empowerment, the current study also explored the decision making process about the right to decide the use of contraceptive. Most women considered it natural to involve their partner in the decision to use EC. This finding is in line with the literature on contraceptive decision making.^22^

The findings of the present study reveal some important policy implications and are useful for health authorities to take up the challenge of expanding the reproductive health services and improving the quality of modern contraception and information delivery systems within Saudi Arabia.

### Limitations

The relatively small number of participants in this study prohibited meaningful statistical analysis. It was conducted on women attending a single tertiary care hospital, which may not be a representative group of women from the community. This reduces the generalizability of the findings. Due to the sensitive nature of the topic, some women were hesitant to disclose their opinions and several forms had to be excluded due to incomplete information. Thus, due to small number the results could not be specifically stratified according to the age groups. Additionally, the questionnaire in the current study did not explore the ideas and concerns regarding the use of EC in depth. A larger sample size and a broader geographical distribution are recommended for further studies on this important reproductive health subject.

## CONCLUSION

The findings of the present study reveal very low levels of knowledge and practice of emergency contraception among married women. Health care professionals were the least reported source of information, which is a cause for concern. The major barriers identified were concerns of women about the possible side effects of EC and its health consequences. For further, clarification qualitative studies are needed to explore in-depth the misconceptions.

## RECOMMENDATIONS

Health care professionals should be encouraged to provide appropriate counseling services related to reproductive health in their consultations tailored to the country level characteristics, in light of the social norms and religious values.

### Implication statement

The findings of the present study reveal some important policy implications and are useful for health authorities to take up the challenge of expanding the reproductive health services and improving the quality of modern contraception and related information delivery systems within Saudi Arabia
